# Comparison of smoking behaviors and associated factors between HIV-infected and uninfected men in Guilin, China: a case–control study

**DOI:** 10.3389/fpsyg.2024.1422144

**Published:** 2024-12-24

**Authors:** Yong Yu, Fuqun Xiao, Mengqiu Xia, Liman Huang, Xiaohu Liu, Wenyu Tang, Xue Gong

**Affiliations:** ^1^School of Politics and Public Administration, Guangxi Normal University, Guilin, China; ^2^Western Urban and Rural Integration Development Institute, Guilin, China; ^3^Guilin Tobacco Company of Guangxi Zhuang Autonomous Region, Guilin, China; ^4^Department of Infectious Diseases, The Third People's Hospital of Guilin City, Guilin, China

**Keywords:** men, smoking, HIV infection, case–control study, China

## Abstract

**Background:**

Smoking is highly prevalent among HIV-infected individuals and is associated with high morbidity and mortality. Studies on smoking among HIV-infected individuals in China, especially compared to uninfected individuals, are scarce.

**Purpose:**

This study aimed to investigate and compare the prevalence and factors associated with smoking between HIV-infected and uninfected men in Guilin, China.

**Methods:**

A survey was conducted among 1,395 HIV-infected men at Guilin Third People’s Hospital from June to December 2022, with a 1:2 age (±1 year) and education-matched control group of 2,790 HIV-uninfected men, whose data were collected from March to August 2023. Both groups completed an online questionnaire on smoking behaviors and relevant characteristics, with a comparative analysis of associated factors conducted using chi-square tests and multivariable logistic regressions.

**Results:**

The rates of former smokers were comparable between HIV-infected and uninfected men [12.3% (95% CI: 10.7–13.9%) vs. 12.0% (95% CI: 10.9–13.1%)], but the current smoking rate was significantly higher in the infected group than in the uninfected group [37.6% (95% CI: 35.2–40.0%) vs. 27.6% (95% CI: 25.9–29.3%), *p* < 0.05]. In both groups, the following factors were significantly associated with smoking: higher income, presence of chronic diseases, negative coping styles, lower social support, and having depression, anxiety, and suicidal ideation. Additionally, in HIV-infected men, the following factors were exclusively associated with smoking: heterosexual HIV transmission route, lower CD4+ T cell count, longer duration of antiretroviral therapy (ART), and drug use.

**Conclusion:**

HIV-infected men had higher smoking rates than their uninfected counterparts, indicating that HIV diagnosis may be a critical timing to initiate behavioral changes and deliver smoking cessation interventions. Furthermore, multiple demographic, clinical, and psychosocial factors were associated with smoking, indicating the need to develop and implement comprehensive smoking cessation prevention and intervention programs.

## Introduction

HIV is a significant global health challenge, having affected approximately 88.4 million people and resulting in around 42.3 million deaths worldwide since the start of the epidemic till now (UNAIDS, 2024). In China, as of June 30, 2024, HIV has affected 1,329,127 individuals and caused 474,006 cumulative deaths (Chinese Center for Disease Control and Prevention, Center for AIDS/STD Prevention and Control, 2024). Although ART has drastically decreased AIDS-associated morbidity and mortality, smoking has gained increasing importance as a significant contributor to excess non-AIDS morbidity and mortality among people living with HIV (Boyer et al., 2023; Ale et al., 2021; Chang et al., 2020).

Accumulating evidence has consistently shown a high prevalence of smoking among HIV-infected individuals (Johnston et al., 2021). For instance, the smoking rate was reported to be 49.4% among HIV-infected individuals in Germany and Austria (Degen et al., 2014), 23.2% in an HIV-infected Asian regional cohort (Do et al., 2016), and 62.0% among HIV-infected men in Yunnan, China (Luo et al., 2014). A recent review and meta-analysis showed that the pooled odds of smoking were 1.64 times greater in HIV-infected individuals than uninfected individuals, and the results stayed similar in sub-group analyses of males, females, and in four of five WHO regions (Johnston et al., 2021). However, recent research indicates a declining trend in smoking among the HIV-infected population. Tochman et al. (2023) followed a cohort of HIV-infected individuals from 2009 to 2019 and observed a statistically significant reduction in the smoking rate, though it was higher than the general population. Wang et al. (2016) study also showed that a large proportion of smoking HIV-infected individuals reduced or quit smoking after HIV diagnosis. Notably, a recent research in Taizhou, China, showed a significantly lower smoking rate among HIV-infected men than among uninfected men (33.9% vs. 46.3%), which is contrary to most previous study findings (Ning et al., 2019). Thus, more research is needed to investigate and compare the smoking behaviors between HIV-infected and uninfected individuals.

HIV-infected smokers are at an increased risk of both AIDS-related and non-AIDS health issues. Studies indicate that smoking may adversely affect ART response; HIV-infected smokers are less likely to achieve an undetectable viral load and a CD4+ T cell lymphocyte recovery of ≥50 cells/μL within six months of initiating ART (McClean et al., 2022). Smoking in the HIV-infected population is also linked to an increased risk of cardiovascular disease, cancer, neurodegenerative disorders, and cognitive deficits compared to those not infected with HIV (Lorenz et al., 2021; Verheij et al., 2021; Liang et al., 2022). For instance, Reddy et al. (2017) study illustrated that HIV-infected smokers were 6–13 times more likely to succumb to lung cancer than to AIDS-related complications. Furthermore, HIV-infected smokers tend to encounter more challenges in quitting smoking compared to the general population (Regan et al., 2016). Therefore, it is critical to explore risk factors of smoking specific to HIV-infected individuals to guide targeted interventions for smoking prevention and cessation.

Previous studies have identified a wide range of factors that contribute to the increased risk of smoking among HIV-infected individuals, among which mental health problems are most cited. HIV infection is associated with significant mental health problems such as depression and anxiety, and HIV-infected individuals may use smoking to cope with emotional challenges (Chang et al., 2017; Weinberger et al., 2017). HIV-infected individuals face multiple health challenges, including frailty, immune deficiency, and fatal complications, which can trigger psychological distress (Rubin and Maki, 2019; Rezaei et al., 2019). HIV-related stigma and discrimination can lead to social rejection and isolation, intensifying mental health issues like depression, anxiety, and suicidal thoughts (Yu et al., 2023). Additionally, long-term ART and side effects, employment and financial strains, and adverse life events present further challenges, driving HIV-infected individuals toward unhealthy behaviors such as smoking (Brandt et al., 2017; Crockett et al., 2018). The impact of these factors on the smoking habits of HIV-infected men requires further exploration.

In summary, investigating the prevalence and risk factors of smoking among HIV-infected individuals is essential to optimize programs and policies for smoking prevention and control and thus reduce non-AIDS morbidity and mortality. Presently, studies on smoking among HIV-infected individuals in China, especially compared to uninfected individuals, are scarce. This study aimed to investigate the prevalence and factors associated with smoking among HIV-infected men as compared to uninfected men in Guilin, Guangxi, China. Our findings will contribute to the development and implementation of smoking prevention and control strategies tailored to the complex needs of HIV-infected individuals.

## Materials and methods

### Participants and procedure

*HIV-infected men* HIV-infected men were recruited from the Department of Infectious Diseases of the Guilin Third People’s Hospital in the Guangxi Zhuang Autonomous Region. Renowned as the leading center for HIV ART in Guilin, the hospital plays a pivotal role in HIV prevention and quality care for the populous northern Guangxi region. The inclusion criteria were as follows: (1) with a confirmed HIV diagnosis, (2) male gender, (3) age between 18 and 65 years, (4) ongoing ART, and (5) willingness to participate in the study with informed consent. We excluded individuals who were unable to participate in the survey due to severe physical or mental health conditions, including those diagnosed with severe depression or other serious mental disorders, as well as those hospitalized for mental health issues within the past six months.

This study was approved by the Ethics Committee of Guangxi Normal University (20220508001), and all participants provided written informed consent before their involvement. Our target sample was all eligible HIV-infected individuals receiving care at the study site from June to December 2022. Potential participants were approached by clinic nurses and referred to our research team for further questionnaire surveys. Owing to the survey’s sensitive nature, data collection was exclusively conducted via Sojump (http://www.sojump.com). Sojump is one of the largest online survey platforms in China, and it provides professional WeChat-based research services, including questionnaire design and distribution and data collection and analysis (Zhang et al., 2017). After providing written informed consent, participants were provided iPads and independently completed the online questionnaire via Sojump. Participants completed the questionnaire in designated offices without interruption to ensure data anonymity and authenticity. Among 1,462 eligible HIV-infected individuals, 1,416 consented to participate, among whom 21 withdrew during the study, resulting in 1,395 valid questionnaires (response rate: 95.4%).

*HIV-uninfected men* In this study, the control group was matched at a 1:2 ratio based on age (±1 year) and educational level, and all participants were required to be residents of the six urban districts and ten counties under the jurisdiction of Guilin. Individuals with severe health conditions that might hinder their participation were excluded, including a diagnosis of major depressive disorder or other significant mental health disorders, as well as recent hospitalization for mental health issues within the past six months. The survey team comprised 120 students from the 2020 and 2021 classes of the School of Politics and Public Administration at Guangxi Normal University. This survey was conducted as part of the practical training in the “Social Survey and Research Methods” course after standardized training. Each student was assigned a list of 12 HIV-infected individuals and was tasked with successfully matching 24 HIV-uninfected individuals (each HIV-infected individual was matched twice) to complete the survey and earn course credits. Data for the control group were collected from June 2022 to March 2023 using the Sojump platform. Written informed consent was obtained, and participants independently completed an online questionnaire via Sojump. Ultimately, valid responses were obtained from 2,790 HIV-negative men.

### Measures

Basic characteristics For both groups, we collected basic demographic characteristics, including age, residence type, ethnicity, marital status, education level, occupation, monthly income, and comorbidity with other chronic diseases, such as hypertension, diabetes, coronary heart disease, hyperlipidemia, chronic obstructive pulmonary disease (COPD), and other conditions requiring long-term maintenance treatment. For HIV-infected men, we additionally collected the following HIV-related disease characteristics: the mode of HIV transmission, the most recent CD4+ T cell count, and the duration of ART. Moreover, we collected information on drug use over the past six months, including methamphetamine (METH), Magu, cannabis, ecstasy, toppinge-cigarettes, ketamine, happy water, and various psychoactive substances.

**Figure 1:**
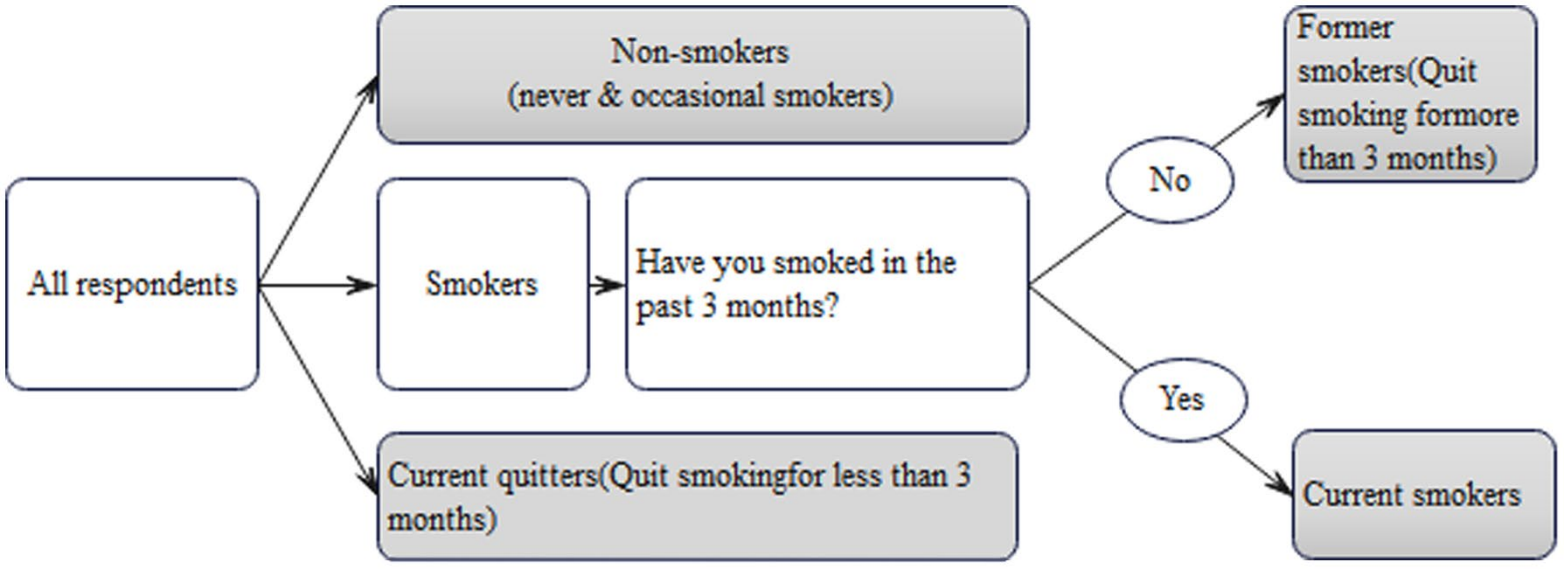
Diagram of non-smokers, current smokers and former smokers indicators.

Measurement and classification of smoking Based on previous studies (Müssener et al., 2020; Kale et al., 2015; Graham et al., 2020), this study used “3 months” as a significant time milestone. For instance, “sustained smoking cessation for 3 months or longer” was regarded as an indicator of successful quitting, while “continuing to smoke for 3 months or longer” signified long-term smoking. All respondents were asked the following three questions: “Are you a smoker?,” “Have you smoked in the past 3 months?” and “How many cigarettes do you smoke daily in the past 3 months?” HIV-infected men were specifically inquired about their smoking cessation after HIV diagnosis and the duration of their abstinence. In contrast, uninfected men were asked about their successful quitting and the length of time they had remained smoke-free. This study followed the Standardized Smoking Survey Guidelines by the World Health Organization (Palipudi et al., 2016), which categorizes smoking status as non-smokers, current smokers, and former smokers, each described below (Figure 1):

- Non-Smokers: This category encompasses two distinct groups. The first group consists of individuals who have never smoked with no history or habit of smoking. The second group includes rare or occasional social smokers who typically smoke infrequently without developing a sustained nicotine dependence.
- Former smokers refer to those who had a regular smoking habit for an extended duration, usually spanning multiple years, and have successfully quit smoking for at least 3 months. For HIV-infected men in this study, former smokers are defined as those who ceased smoking for over 3 months following their HIV diagnosis.
- Current smokers are individuals who have continued smoking over a long period (at least 3 months) and smoke daily, typically consuming at least half a pack or more each day.

Coping style Coping was assessed using the Simplified Coping Style Questionnaire (SCSQ) developed by Xie (1998), designed to evaluate various coping styles individuals use when facing life stress and difficulties. The SCSQ comprises 20 items under two domains: negative and positive coping. A coping tendency score is calculated as the score difference of positive minus negative coping score (Xie, 1998), with a positive score indicating a propensity for more positive coping in response to stress and vice versa. In this study, the Cronbach’s *α* coefficient for the SCSQ among HIV-infected and uninfected individuals was recorded at 0.86 and 0.89, respectively.

Social support Social support was assessed using the Perceived Social Support Scale (PSSS) developed by Xiao (1994). The PSSS comprises 12 items under three dimensions: family support, friend support, and other forms of support. It utilizes a seven-point scoring system (ranging from 1 to 7), with the total score ranging from 12 to 84, and cutoffs 36 and 60 distinguishing between low (12–36), medium (37–60), and high social support (61–84). In this research, Cronbach’s α coefficient for the PSSS was 0.94 among HIV-infected patients and 0.90 in uninfected individuals.

Depression, anxiety, and stress Depression, anxiety, and stress were evaluated by the Depression, Anxiety, and Stress Scale (DASS-21) (Wang et al., 2016). It includes 21 items evenly divided into three subscales for depression, anxiety, and stress. Each item is rated on a four-point scale from “0” (not applicable) to “3” (very applicable), and each subscale score ranges from 0 to 21, with a higher score representing a higher level of depression, anxiety, and stress, respectively. In this study, a cutoff of 7 was used to distinguish between those with and without depression, anxiety, and stress. The Cronbach’s alpha coefficients for the DASS-21 among HIV-infected men and uninfected men were 0.89 and 0.91, respectively.

Suicidal ideation Suicide ideation was evaluated using two items from the Beck Scale for Suicide Ideation-Chinese Version (BSI-CV) (Li et al., 2011). The first item assesses the respondent’s active desire to attempt suicide, and the second item assesses passive suicidal desire (e.g., wishing to remain asleep, die unexpectedly, etc.). Participants who responded with ‘weak’ or ‘moderate to strong’ to either of these items were classified as exhibiting suicidal ideation.

### Statistical analyses

All statistical analyses were conducted using SPSS 26.0 software. Continuous variables were presented as means (standard deviations), while categorical variables were reported as frequencies and percentages. Normality tests were conducted for all continuous variables using the Shapiro–Wilk test, which confirmed the normal distribution of these variables. Comparisons of sample characteristics between the HIV-infected and uninfected groups were conducted using Pearson’s chi-square test. Additionally, four multivariable logistic regression models were utilized to examine factors associated with past and current smoking among both groups. To further compare smoking behaviors between HIV-infected and uninfected men, two additional multivariable logistic regression models were used, treating HIV status as the primary exposure factor and controlling for key demographic and psychosocial variables. The independent variables were selected based on the following criteria: (1) Variables with *p* values >0.20 in the univariable analysis, or (2) variables that were well-established risk factors in the literature, such as age, residential type, and nationality. *p*-values less than 0.05 were considered statistically significant.

## Results

### Group comparison of sample characteristics

In this study, 1,395 HIV-infected and 2,790 uninfected men were enrolled, with average ages of 35.5 (11.1) and 35.6 (11.1) years, respectively. Both groups had the same proportion of participants with a bachelor’s degree or higher (50.5%). For other sociodemographic characteristics, the two groups showed statistically significant differences in resident type and occupation. Compared to the uninfected group, the HIV-infected group was more likely to be employed and live in urban areas. See Table 1 for details.

**Table 1:** Comparison of sample characteristics of HIV-infected men and HIV-uninfected men.

Variables	HIV-infected men (*n* = 1,395)	HIV-uninfected men (*n* = 2,790)	χ^2^/*t*	*P*-values
Age	35.5 (11.1)	35.6 (11.1)		
Education level				
Below bachelor degree	691 (49.5)	1,382 (49.5)	0.00	1.000
Bachelor’s degree or above	704 (50.5)	1,408 (50.5)		
Resident type				
Urban	796 (57.1)	1,269 (45.5)	49.87	<0.001
Rural	599 (42.9)	1,521 (54.5)		
Nationality				
Han nationality	1,151 (82.5)	2,260 (81.0)	1.40	0.237
Minority nationality	244 (17.5)	530 (19.0)		
Marital status				
Unmarried	536 (38.4)	982 (35.2)	4.47	0.107
Married	819 (58.7)	1732 (62.1)		
Divorced/widowed	40 (2.9)	76 (2.7)		
Occupation				
Student/unemployment	101 (7.2)	433 (15.5)	100.79	<0.001
employment	1,294 (92.8)	2,357 (84.5)		
Monthly income/RMB				
<4,000	566 (40.6)	1,152 (41.3)	0.20	0.657
≥4,000	829 (59.4)	1,638 (58.7)		
Coping style tendency				
Negative	331 (23.7)	583 (20.9)	6.14	0.046
Positive = negative	346 (24.8)	770 (27.6)		
Positive	718 (51.5)	1,437 (51.5)		
Social support				
Low	278 (19.9)	503 (18.0)	39.80	<0.001
Medium	746 (53.5)	1,275 (45.7)		
High	371 (26.6)	1,012 (36.3)		
Depression				
No	862 (61.8)	2,214 (79.4)	147.28	<0.001
Yes	533 (38.2)	576 (20.6)		
Anxiety				
No	967 (69.3)	2,149 (77.0)	29.04	<0.001
Yes	428 (30.7)	641 (23.0)		
Stress				
Low	934 (67.0)	2023 (72.5)	13.85	<0.001
High	461 (33.0)	767 (27.5)		
Suicidal ideation				
No	855 (61.3)	2,289 (82.0)	214.33	<0.001
Yes	540 (38.7)	501 (18.0)		
Have other chronic diseases				
No	1,041 (74.6)	2,259 (81.0)	22.45	<0.001
Yes	354 (25.4)	531 (19.0)		
Smoking status				
Non-smoking	698 (50.0)	1,686 (60.4)	48.01	<0.001
Former smoking	172 (12.3)	334 (12.0)		
Current smoking	525 (37.6)	770 (27.6)		

For clinical characteristics, the HIV-infected group was more likely to have comorbidities than the uninfected group (25.4 vs. 19.0%, *p* < 0.001). Most of the HIV-infected group contracted HIV through the heterosexual route (72.0%), had been on ART for over 3 years (62.7%), and had CD4+ T cell count≥350/μl (61.2%), which are not presented in Table 1. Regarding psychosocial characteristics, the HIV-infected group was less likely to have high social support (26.6% vs. 36.6%, *p* < 0.001) and more likely to have depression (38.2% vs. 20.5%, *p* < 0.001), anxiety (30.7% vs. 23.0%, *p* < 0.001), stress (33.0% vs. 27.5%, *p* < 0.001) and suicidal ideation (38.7% vs. 18.0%, *p* < 0.001) than the uninfected group. See Table 1 for details.

### Group comparison of smoking behaviors

In this study, the current smoking rate was significantly higher in the HIV-infected group (37.6%; 95% CI: 35.2–40.0%) than in the uninfected group (27.6%; 95% CI: 25.9–29.3%; *p* < 0.05). In contrast, former smoking rates were comparable between the HIV-infected group (12.3%; 95% CI: 10.7–13.9%) and the uninfected group (12.0, 95% CI: 10.9–13.1%, *p* > 0.05). Among current smokers, the HIV-infected group was more likely to have over ten cigarettes per day than the uninfected group (59.0% vs. 50.1%, *p <* 0.005) (Results not shown). When comparing group characteristics by smoking status, significant differences were observed in all variables except age, education, place of residence, ethnicity, and social support between the HIV-infected and uninfected groups (Table 2).

**Table 2:** Comparison of sample characteristics between non-smokers, former smokers, and current smokers among HIV-infected men and HIV-uninfected men.

Variables	HIV-infected men (*n* = 1,395)			*χ* ^2^	*P*-values	HIV-uninfected men (*n* = 2,790)			*χ* ^2^	*P*-values
	non-smoking (*n* = 698)	former smoking (*n* = 172)	current smoking (*n* = 525)			non-smoking (*n* = 1,686)	former smoking (*n* = 334)	current smoking (*n* = 770)		
Age (years)										
<36	446 (63.9)	89 (51.7)	310 (59.0)	9.35	0.009	1,013 (60.1)	209 (62.6)	477 (61.9)	1.22	0.543
≥36	252 (36.1)	83 (48.3)	215 (41.0)			673 (39.9)	125 (37.4)	293 (38.1)		
Education level										
Below bachelor degree	294 (42.1)	109 (63.4)	288 (54.9)	34.47	<0.001	839 (49.8)	183 (54.8)	360 (46.8)	6.11	0.047
Bachelor’s degree or above	404 (57.9)	63 (36.6)	237 (45.1)			847 (50.2)	151 (45.2)	410 (53.2)		
Resident type										
Urban	389 (55.7)	97 (56.4)	310 (59.0)	1.38	0.510	764 (45.3)	162 (48.5)	343 (44.5)	1.52	0.467
Rural	309 (44.3)	75 (43.6)	215 (41.0)			922 (54.7)	172 (51.5)	427 (55.5)		
Nationality										
Han nationality	569 (81.5)	135 (78.5)	447 (85.1)	4.93	0.085	1,353 (80.2)	269 (80.5)	638 (82.9)	2.39	0.303
Minority nationality	129 (18.5)	37 (21.5)	78 (14.9)			333 (19.8)	65 (19.5)	132 (17.1)		
Marital status										
Unmarried	312 (44.7)	66 (38.4)	158 (30.1)	27.47	<0.001	636 (37.7)	90 (26.9)	256 (33.2)	19.01	<0.001
Married	370 (53.0)	101 (58.7)	348 (66.3)			1,006 (59.7)	238 (71.3)	488 (63.4)		
Divorced/widowed	16 (2.3)	5 (2.9)	19 (3.6)			44 (2.6)	6 (1.8)	26 (3.4)		
Occupation										
Student/unemployment	60 (8.6)	18 (20.5)	23 (4.4)	10.97	0.004	297 (17.6)	45 (13.5)	91 (11.8)	14.76	0.001
employment	638 (91.4)	154 (89.5)	502 (95.6)			1,389 (82.4)	289 (86.5)	679 (88.2)		
Monthly income/RMB										
<4,000	329 (47.1)	68 (39.5)	169 (32.2)	27.84	<0.001	772 (45.8)	105 (31.4)	275 (35.7)	37.33	<0.001
≥4,000	369 (52.9)	104 (60.5)	356 (67.8)			914 (55.8)	229 (68.6)	495 (64.3)		
Coping style tendency										
Negative	126 (18.1)	45 (26.2)	160 (30.5)	31.83	<0.001	261 (15.5)	119 (35.6)	203 (26.4)	105.03	<0.001
Positive = negative	168 (24.1)	47 (27.3)	131 (25.0)			455 (27.0)	98 (29.3)	217 (28.2)		
Positive	404 (57.9)	80 (19.8)	234 (44.6)			970 (57.5)	117 (35.0)	350 (45.5)		
Social support										
low	119 (17.0)	42 (24.4)	117 (22.2)	8.15	0.086	239 (14.2)	95 (28.4)	169 (21.9)	72.38	<0.001
medium	381 (54.6)	88 (51.2)	277 (52.8)			755 (44.8)	161 (48.2)	359 (46.6)		
high	198 (28.4)	42 (24.4)	131 (25.0)			692 (41.0)	78 (23.4)	242 (31.4)		
Depression										
No	494 (70.8)	785 (49.4)	283 (53.9)	48.84	<0.001	1,436 (85.2)	243 (72.8)	535 (69.5)	89.53	<0.001
Yes	204 (29.2)	87 (50.6)	242 (46.1)			250 (14.8)	91 (27.2)	235 (30.5)		
Anxiety										
No	518 (74.2)	103 (59.9)	346 (65.9)	17.94	<0.001	1,392 (82.6)	216 (64.7)	541 (70.3)	77.93	<0.001
Yes	180 (25.8)	69 (40.1)	179 (34.1)			294 (17.4)	118 (35.3)	229 (29.7)		
Stress										
Low	514 (73.6)	104 (60.5)	316 (60.2)	28.23	<0.001	1,345 (79.8)	204 (61.1)	474 (61.6)	112.87	<0.001
High	184 (26.4)	68 (39.5)	209 (39.8)			341 (20.2)	130 (38.9)	296 (38.4)		
Suicidal ideation										
No	468 (67.0)	97 (56.4)	290 (55.2)	19.60	<0.001	1,468 (87.1)	248 (74.3)	573 (74.4)	73.09	<0.001
Yes	230 (33.0)	75 (43.6)	235 (44.8)			218 (12.9)	86 (25.7)	197 (25.6)		
The route of infection with HIV										
Male–male	257 (36.8)	38 (22.1)	95 (18.1)	55.51	<0.001	—	—	—	—	—
Heterosexual	441 (63.2)	134 (77.9)	430 (81.9)			—	—	—	—	—
CD4+ T cell count										
≥350/μl	473 (67.8)	93 (54.1)	288 (54.9)	25.25	<0.001	—	—	—	—	—
<350/μl	225 (32.2)	79 (45.9)	237 (45.1)			—	—	—	—	—
ART duration										
<3 years	305 (43.7)	53 (30.8)	162 (30.9)	24.63	<0.001	—	—	—	—	—
≥3 years	393 (56.3)	119 (69.2)	363 (69.1)			—	—	—	—	—
Have other chronic diseases										
No	553 (79.2)	123 (71.5)	365 (69.5)	15.90	<0.001	1,455 (86.3)	227 (68.0)	577 (74.9)	85.93	<0.001
Yes	145 (20.8)	49 (28.5)	160 (30.5)			231 (13.7)	107 (32.0)	193 (25.1)		
Drug use										
No	670 (96.0)	155 (90.1)	475 (90.5)	17.73	<0.001	—	—	—	—	—
Yes	28 (4.0)	17 (9.9)	50 (9.5)			—	—	—	—	—

### Factors associated with smoking behaviors

Table 3 presents the outcomes of four logistic regression models analyzing factors associated with current and former smoking behaviors among HIV-infected and uninfected men. Among HIV-infected men, former smoking was associated with being aged ≥36 years (aOR = 1.57, 95% CI: 1.05–2.36), having higher income (≥4,000 RMB) (aOR = 1.21, 95% CI: 0.82–1.78), experiencing depression (aOR = 1.90, 95% CI: 1.26–2.87), having suicidal ideation (aOR = 1.53, 95% CI: 1.05–2.21), heterosexual transmission (aOR = 1.74, 95% CI: 1.10–2.75), and a CD4+ T cell count <350/μl (aOR = 1.55, 95% CI: 1.08–2.25). For current smoking, factors associated with a higher odds included being married (aOR = 1.50, 95% CI: 1.11–2.03), having higher income (≥4,000 RMB) (aOR = 1.67, 95% CI: 1.28–2.18), experiencing depression (aOR = 1.63, 95% CI: 1.22–2.17), having suicidal ideation (aOR = 1.53, 95% CI: 1.18–1.99), heterosexual transmission (aOR = 1.96, 95% CI: 1.43–2.66), CD4+ T cell count <350/μl (aOR = 1.54, 95% CI: 1.20–1.99), ART duration ≥3 years (aOR = 1.38, 95% CI: 1.06–1.81), presence of chronic diseases (aOR = 1.35, 95% CI: 1.01–1.80), and drug use (aOR = 1.76, 95% CI: 1.05–2.96). Conversely, an education level below a bachelor’s degree (aOR = 0.44, 95% CI: 0.30–0.64) and being employed (aOR = 0.44, 95% CI: 0.22–0.86) were associated with a lower odds of former smoking, while negative coping strategies were associated with a lower odds of current smoking (aOR = 0.57, 95% CI: 0.42–0.79).

**Table 3:** Multivariable logistic regression analysis of former and current smoking in HIV-infected and non-HIV-infected men.

Variables	HIV-infected men				HIV-uninfected men			
	Former smoking (Model 3)		Current smoking (Model 4)		Former smoking (Model 5)		Current smoking (Model 6)	
	aOR(95%CI)	*P*-values	aOR(95%CI)	*P*-values	aOR(95%CI)	*P*-values	aOR(95%CI)	*P*-values
Age (years)								
<36	1.00		1.00		1.00		1.00	
≥36	1.57 (1.05 ~ 2.36)	0.029	0.88 (0.67 ~ 1.17)	0.400	0.88 (0.68 ~ 1.14)	0.330	0.93 (0.78 ~ 1.12)	0.461
Education level								
Below bachelor degree	1.00		1.00		1.00		1.00	
Bachelor’s degree or above	0.44 (0.30 ~ 0.64)	<0.001	0.69 (0.53 ~ 0.89)	0.004	0.83 (0.64 ~ 1.07)	0.144	1.15 (0.96 ~ 1.37)	0.142
Resident type								
Urban	1.00		1.00		1.00		1.00	
Rural	0.95 (0.65 ~ 1.38)	0.771	0.94 (0.73 ~ 1.21)	0.625	0.86 (0.67 ~ 1.11)	0.247	1.02 (0.85 ~ 1.23)	0.875
Nationality								
Han nationality	1.00		1.00		1.00		1.00	
Minority nationality	1.30 (0.83 ~ 2.01)	0.251	0.80 (0.58 ~ 1.12)	0.203	1.02 (0.74 ~ 1.40)	0.920	0.87 (0.69 ~ 1.10)	0.237
Marital status								
Unmarried	1.00		1.00		1.00		1.00	
Married	1.09 (0.69 ~ 1.71)	0.719	1.50 (1.11 ~ 2.03)	0.009	1.31 (0.94 ~ 1.84)	0.113	0.91 (0.73 ~ 1.14)	0.405
Divorced/widowed	0.88 (0.27 ~ 2.92)	0.834	1.79 (0.81 ~ 3.93)	0.150	0.56 (0.21 ~ 1.49)	0.244	1.06 (0.61 ~ 1.84)	0.844
Occupation								
Student/unemployment	1.00		1.00		1.00		1.00	
Employment	0.44 (0.22 ~ 0.86)	0.017	1.00 (0.57 ~ 1.77)	0.994	0.66 (0.41 ~ 1.07)	0.092	1.40 (1.01 ~ 1.94)	0.041
Monthly income/RMB								
<4,000	1.00		1.00		1.00		1.00	
≥4,000	1.21 (0.82 ~ 1.78)	0.025	1.67 (1.28 ~ 2.18)	<0.001	1.69 (1.23 ~ 2.31)	0.001	1.27 (1.03 ~ 1.56)	0.025
Coping style tendency								
Negative	1.00		1.00		1.00		1.00	
Positive = negative	0.91 (0.54 ~ 1.53)	0.726	0.70 (0.49 ~ 1.01)	0.056	0.49 (0.35 ~ 0.68)	<0.001	0.73 (0.56 ~ 0.94)	0.015
Positive	0.80 (0.50 ~ 1.27)	0.334	0.57 (0.42 ~ 0.79)	0.001	0.37 (0.27 ~ 0.51)	<0.001	0.70 (0.55 ~ 0.89)	0.003
Social support								
Low	1.00		1.00		1.00		1.00	
Medium	0.72 (0.45 ~ 1.15)	0.169	0.73 (0.53 ~ 1.02)	0.068	0.52 (0.38 ~ 0.71)	<0.001	0.67 (0.52 ~ 0.86)	0.002
High	0.72 (0.42 ~ 1.24)	0.234	0.75 (0.51 ~ 1.10)	0.138	0.33 (0.23 ~ 0.47)	<0.001	0.54 (0.41 ~ 0.71)	<0.001
Depression								
No	1.00		1.00		1.00		1.00	
Yes	1.90 (1.26 ~ 2.87)	0.002	1.63 (1.22 ~ 2.17)	0.001	1.07 (0.76 ~ 1.51)	0.710	1.52 (1.15 ~ 2.02)	0.004
Anxiety								
No	1.00		1.00		1.00		1.00	
Yes	1.14 (0.74 ~ 1.78)	0.551	0.77 (0.56 ~ 1.07)	0.117	1.70 (1.24 ~ 2.31)	0.001	1.02 (0.78 ~ 1.33)	0.911
Stress								
Low	1.00		1.00		1.00		1.00	
High	1.06 (0.69 ~ 1.62)	0.809	1.35 (1.00 ~ 1.83)	0.050	1.49 (1.08 ~ 2.06)	0.016	1.65 (1.25 ~ 2.18)	0.001
Suicidal ideation								
No	1.00		1.00		1.00		1.00	
Yes	1.53 (1.05 ~ 2.21)	0.026	1.53 (1.18 ~ 1.99)	0.001	1.62 (1.19 ~ 2.21)	0.002	1.66 (1.31 ~ 2.09)	<0.001
The route of infection with HIV								
Male–male	1.00		1.00		—	—	—	—
Heterosexual	1.74 (1.10 ~ 2.75)	0.018	1.96 (1.43 ~ 2.66)	<0.001	—	—	—	—
CD4+ T cell count								
≥350/μl	1.00		1.00		—	—	—	—
<350/μl	1.55 (1.08 ~ 2.25)	0.018	1.54 (1.20 ~ 1.99)	0.001	—	—	—	—
ART duration								
<3 years	1.00		1.00		—	—	—	—
≥3 years	1.25 (0.84 ~ 1.87)	0.264	1.38 (1.06 ~ 1.81)	0.017	—	—	—	—
Have other chronic diseases								
No	1.00		1.00		1.00		1.00	
Yes	1.24 (0.82 ~ 1.87)	0.317	1.35 (1.01 ~ 1.80)	0.042	2.31 (1.71 ~ 3.11)	<0.001	1.90 (1.51 ~ 2.39)	<0.001
Drug use								
No	1.00		1.00		—	—	—	—
Yes	1.89 (0.96 ~ 3.72)	0.065	1.76 (1.05 ~ 2.96)	0.033	—	—	—	—

Among uninfected men, former smoking was associated with higher income (≥4,000 RMB) (aOR = 1.69, 95% CI: 1.23–2.31), anxiety (aOR = 1.70, 95% CI: 1.24–2.31), high stress (aOR = 1.49, 95% CI: 1.08–2.06), suicidal ideation (aOR = 1.62, 95% CI: 1.19–2.21), and presence of chronic diseases (aOR = 2.31, 95% CI: 1.71–3.11). For current smoking, factors associated with higher odds included being employed (aOR = 1.40, 95% CI: 1.01–1.94), higher income (≥4,000 RMB) (aOR = 1.27, 95% CI: 1.03–1.56), depression (aOR = 1.52, 95% CI: 1.15–2.02), high stress (aOR = 1.65, 95% CI: 1.25–2.18), suicidal ideation (aOR = 1.66, 95% CI: 1.31–2.09), and chronic diseases (aOR = 1.90, 95% CI: 1.51–2.39). Conversely, factors associated with a lower odds of former smoking included negative coping strategies (aOR = 0.49, 95% CI: 0.35–0.68), moderate social support (aOR = 0.52, 95% CI: 0.38–0.71), and high social support (aOR = 0.33, 95% CI: 0.23–0.47), while for current smoking, a lower odds was associated with moderate social support (aOR = 0.67, 95% CI: 0.52–0.86), high social support (aOR = 0.54, 95% CI: 0.41–0.71), and negative coping strategies (aOR = 0.73, 95% CI: 0.56–0.94).

In summary, depression, suicidal ideation, income, coping, social support, and chronic diseases were common factors associated with both former and current smoking behaviors among HIV-infected and uninfected groups. Additionally, in HIV-infected men, the following factors were exclusively associated with smoking: HIV transmission route, CD4+ T cell count, duration of antiretroviral therapy (ART), and drug use.

### HIV and smoking

Table 4 presents two multivariable regression models exploring the association between HIV status (exposure variable) and smoking status (outcome variable). In these models, smoking was assessed as either current or past versus never while controlling for key parameters such as age, education level, residence type, ethnicity, marital status, occupation, monthly income, coping style, social support, depression, anxiety, stress, and suicidal ideation. HIV-specific parameters were excluded to ensure consistency with the control group. The results showed that HIV-infected men had higher odds of current smoking compared to uninfected men (aOR = 1.28, 95% CI: 1.10–1.50, *p* = 0.002), whereas no significant association was found for former smoking (aOR = 1.06, 95% CI: 0.84–1.33, *p* = 0.638).

**Table 4:** Multivariable logistic regression models of HIV status and smoking behaviors (former and current) in HIV-infected and uninfected men^a,b^.

	Dependent variables	Independent variables		aOR(95%CI)	*P*-values
Model 1	Former smoking	HIV	Yes	1.06 (0.84 ~ 1.33)	0.638
			No	1.00	
Model 2	Current smoking	HIV	Yes	1.28 (1.10 ~ 1.50)	0.002
			No	1.00	

^aThe β coefficients in each regression model were estimated after controlling for the following variables: age, education level, residence type, ethnicity, marital status, occupation, monthly income, coping style tendency, social support, depression, anxiety, stress, and suicidal ideation.^

^bAny HIV-specific parameters were excluded from the regression models as they were not applicable to the control group.^

## Discussion

### Summary of the findings

In the large group comparison of HIV-infected and uninfected men, we observed a significantly higher current smoking rate in HIV-infected men compared to their uninfected counterparts. In both groups, a variety of demographic factors (e.g., higher income associated with a higher odds of smoking), clinical factors (e.g., presence of comorbidities), and psychosocial factors (e.g., lower social support, negative coping style, depression, anxiety, and suicidal ideation) were associated with smoking. In the HIV-infected group, factors such as age, educational level, and marital status were associated with smoking behaviors. In the uninfected group, employment status and stress levels were associated with smoking behaviors. Additionally, among HIV-infected men, HIV-related clinical characteristics—including heterosexual mode of HIV transmission, longer ART duration, CD4+ T cell count <350/μl, and history of drug use—were also associated with smoking behavior. These findings provide novel insights into the prevalence and correlates of smoking in HIV-infected versus uninfected men, underscoring the need for targeted and comprehensive smoking cessation programs tailored to the unique profiles of HIV-infected men.

### Smoking among HIV-infected men

This research indicated a notably higher smoking rate in HIV-infected men than in uninfected counterparts, consistent with some global studies. For example, a U.S. study showed that 40.9% of HIV-infected men smoked, contrasting with 23.3% in the broader male population (Mdodo et al., 2015). In South Africa, 52% of HIV-infected men were smokers, against 31.9% in the general male populace (Elf et al., 2018). These comparisons suggest a higher smoking prevalence among HIV-infected men in China compared to uninfected men, aligning closely with global reports on smoking rates (Johnston et al., 2021).

The higher smoking rates among HIV-infected men compared to uninfected counterparts in our study may be explained by the following possible reasons. The diagnosis of HIV often brings a significant psychological burden, including stress, anxiety, and depression, due to stigma and the chronic nature of the disease. Smoking is frequently adopted as a coping mechanism to alleviate psychological pressure despite its well-known adverse health effects (Brandt et al., 2017). Additionally, the social environments of some HIV-infected individuals, which may include higher rates of substance use and behaviors associated with HIV acquisition, could contribute to the normalization of smoking within these groups, further exacerbating smoking prevalence (Pacek et al., 2014). Moreover, the integration of smoking cessation interventions within HIV care settings is not always prioritized, potentially leading to missed opportunities for smoking cessation support. The focus on HIV management often overshadows the need to address modifiable risk factors such as smoking, which is crucial for improving overall health outcomes (Ledgerwood and Yskes, 2016). One important implication of these findings is that HIV diagnosis may be a critical time point to initiate smoking cessation and other behavioral interventions to improve the health outcomes and well-being of HIV-infected individuals. It is necessary to develop integrated care approaches that address both HIV treatment and broader health behaviors, including smoking cessation, to improve overall health outcomes for HIV-infected individuals.

### Factors associated with smoking in HIV-infected men

The study identified several similar sociodemographic and psychosocial factors between HIV-infected and uninfected men. Firstly, higher income may incur more leisure activities that promote smoking, and economic ease may position smoking as a stress reliever (Siahpush et al., 2007; Widome et al., 2015). Secondly, higher social support indicates more access to emotional and practical aid, which can mitigate smoking behaviors among HIV-infected individuals (Krishnan et al., 2018). Thirdly, negative coping strategies can lead to increased smoking behavior as individuals may use smoking as a means to alleviate stress and emotional distress (Leventhal and Zvolensky, 2015). Lastly, mental health issues, particularly depression, anxiety, and suicidal thoughts, may trigger smoking as a form of self-regulation and self-coping, which was accentuated in those living with HIV due to illness-related stress (Leventhal and Zvolensky, 2015).

The study also identified several distinct sociodemographic and psychosocial factors associated with smoking among HIV-infected and uninfected individuals. For instance, factors such as age, education level, and marital status were associated with smoking risks among HIV-infected individuals. In contrast, employment and stress were correlated with smoking risks among uninfected men. Among HIV-infected men, higher smoking rates are notably observed among those with younger age, lower educational attainment, and disrupted marital status, such as divorced or widowed. These factors suggest that lifestyle challenges and stressors, including limited health literacy and emotional distress from marital instability, may drive the inclination toward smoking as a coping strategy (Park and Iacocca, 2014). Conversely, in uninfected men, employment and stress emerge as significant contributors to smoking behaviors. Employed men may be more likely to smoke due to workplace stress and the social normalization of smoking during breaks or in networking contexts (Saetta et al., 2023). Additionally, men experiencing higher levels of stress may resort to smoking as a coping mechanism to alleviate psychological distress and manage stress-related symptoms (Park and Iacocca, 2014).

In addition, this study identified several influencing factors of smoking that were unique to HIV-infected men, including the route of HIV transmission, the duration of ART, CD4 + T cell counts, and drug use. Notably, heterosexual HIV-infected men showed higher smoking rates than homosexual men, which may be explained by cultural and behavioral differences between these groups. Research indicates that certain social factors associated with sexual orientation and health behaviors may contribute to these differences in smoking rates (Moskowitz and Hart, 2011; Liu et al., 2020). Furthermore, individuals on long-term ART exhibit increased smoking rates, which could be explained by the stabilized health conditions with ongoing treatment, leading to lifestyle and behavioral choices, including smoking. Additionally, lower CD4 + T cell counts were associated with an increased risk of smoking, which may indicate a reverse causal link; that is, smoking could decrease the overall health and immune function of HIV-infected individuals, leading to decreased CD4 + T cell counts, which requires further investigation (Elf et al., 2018). Finally, the positive association between drug use and smoking behavior in HIV-infected men indicates the intersecting epidemics of HIV infection, drug use, and smoking. This finding was consistent with previous studies showing a high coexistence of these problems (Marshall et al., 2011; McCoy et al., 2004; Bhalerao and Cucullo, 2020). The cooccurrence of drug use and smoking in HIV-infected men can be explained by the well-established minority stress associated with each behavior (Amato and Émond, 2023; Ogunbajo et al., 2021). Therefore, mechanisms of maladaptive behaviors, including drug use and smoking, all tend to emerge in a synergistic manner (Bhalerao and Cucullo, 2020).In addition, HIV infection, drug use, and smoking drug users’ predisposition to addiction may confer an enhanced physiological dependence on nicotine, leading to a higher risk of smoking and vice versa (Marshall et al., 2011). Furthermore, drug use and smoking share similar mechanisms in activating the brain’s reward system to achieve satisfaction, which may produce synergistic effects in dual users who concurrently use both drugs and smoking to maximize satisfaction (Pacek et al., 2014).

### Implications

Our study carries significant clinical, research, and policy implications to inform future smoking cessation interventions among HIV-infected men. Healthcare providers should initiate smoking cessation as soon as HIV diagnosis and incorporate it into the broader treatment plans for HIV-infected individuals. Specialized smoking cessation programs tailored to the unique challenges of HIV-infected individuals are needed. Future long-term studies should elucidate the causal links between HIV infection and smoking behavior across diverse geographic and socioeconomic settings. On the policy front, public health strategies and policies are needed to improve smoking cessation awareness and provide accessible cessation resources. Policymakers should consider integrating smoking cessation programs with HIV care services to ensure a holistic approach to patient health.

### Limitations

This study has several limitations. First, the cross-sectional design restricts the ability to establish causality between HIV status and smoking behaviors, and future longitudinal studies are necessary to clarify the temporal dynamics and potential causal relationships. Second, reliance on self-reported data for both smoking and HIV status may introduce reporting bias; in particular, self-reported HIV status in the control group could lead to potential misclassification. Third, the control group was enrolled one year after the HIV-infected group, potentially introducing selection bias, though we anticipate that this time difference has a minimal effect given the long-term nature of smoking behavior. Fourth, our sample was recruited exclusively from a hospital in Guangxi Province and may not represent the wider Chinese or Asian population. However, our major findings were consistent with the wider global literature, indicating our results may be generalizable to a wider population, which warrants further study. Fifth, this study only focused on men, and the results may not be applicable to women due to the significant gender differences in socioeconomic status, lifestyles, and health risk behaviors. Future studies should consider including female participants and test whether the same conclusions hold for females. Additionally, four multivariable logistic regression models were utilized to examine factors associated with past and current smoking among both groups. The independent variables were selected based on the theoretical and empirical evidence showing significant associations between psychosocial factors and smoking. Specifically, for this study, we selected the following psychosocial variables: coping, social support, depression, anxiety, stress, and suicidal ideation. In addition, we included all demographic variables, such as age, education level, residence type, ethnicity, marital status, occupation, and monthly income, to control for their potential confounding effects. Sixth, our study showed significant differences in employment status between the two groups, with more students in the control group, which may affect observed smoking behaviors, as employment can influence smoking habits. However, we have adjusted for employment status in all multivariable analyses to control for its potential confounding effects. Seventh, the study did not collect detailed data on smoking behaviors, such as duration, age at first use, and intensity. Future studies should incorporate these details to provide a more comprehensive understanding of smoking patterns. Finally, our study only included certain psychosocial factors (e.g., coping, social support, depression, anxiety, stress, and suicidal ideation) into our regression model to explore their associations with smoking behaviors. Future studies should consider adding other factors, such as self-esteem, family functioning, and resilience, to get a more comprehensive understanding of the psychosocial correlates of smoking behaviors. Addressing these limitations will enhance understanding of smoking behaviors among HIV-infected individuals and inform targeted interventions.

## Conclusion

Our study showed a higher smoking rate in HIV-infected men than in uninfected men, which is consistent with most previous studies. This finding indicates HIV diagnosis may be a critical timing to initiate behavioral changes and deliver smoking cessation interventions. Furthermore, multiple demographic, clinical, and psychosocial factors were associated with smoking, indicating the need to develop and implement comprehensive smoking cessation prevention and intervention programs based on these factors. Future research should delve into longitudinal studies across varied populations, and policies should focus on integrating smoking cessation programs into HIV care services.

## Data Availability

The raw data supporting the conclusions of this article will be made available by the authors, without undue reservation.
